# Aortic Stenosis: Time for a Sex-Based Approach?

**DOI:** 10.3390/jcm14082691

**Published:** 2025-04-15

**Authors:** Aurelia Zimmerli, Quentin Liabot, Georgios Tzimas, Mariama Akodad, Adil Salihu, Victor Weerts, Panagiotis Antiochos, Stephanie L. Sellers, Pierre Monney, Olivier Muller, Stephane Fournier, David Meier

**Affiliations:** 1 Service of Cardiology, Lausanne University Hospital and University of Lausanne, Rue du Bugnon 46, 1011 Lausanne, Switzerlandquentin.liabot@chuv.ch (Q.L.); victor.weerts@chuv.ch (V.W.); pierre.monney@chuv.ch (P.M.);; 2 Institut Cardiovasculaire Paris-Sud (ICPS), Hôpital Jacques Cartier, Ramsay-Santé, 91300 Massy, France; 3 Dilawri Cardiovascular Institute, Vancouver General Hospital, Vancouver, BC V5Z 1M9, Canada; ssellers@providencehealth.bc.ca; 4 Cardiovascular Translational Laboratory, Providence Research and Centre for Heart Lung Innovation, St Paul’s Hospital and University of British Columbia, Vancouver, BC V6Z 1Y6, Canada

**Keywords:** aortic stenosis, women, TAVI, pathophysiology, sex-specific

## Abstract

Aortic stenosis (AS) is a progressive form of valvular heart disease most commonly associated with aging, with an exponential increase in prevalence after age 50. While men have historically been considered at higher risk, recent studies highlight a similar prevalence between men and women, with a higher prevalence in elderly women driven by longer life expectancy. Sex-related differences in clinical presentation, anatomy, and pathophysiology influence disease progression, severity assessment, and management. Women are often diagnosed at more advanced stages, exhibiting more pronounced symptoms, typically dyspnea and functional impairment, whereas men more often report chest pain. Women have a smaller body surface area, leading to smaller aortic annuli, left ventricular outflow tracts, aortic roots impacting flow dynamic, and severity grading. Diagnostic challenge contributes to the undertreatment of women. Despite experiencing severe AS, women receive fewer interventions and face delays in treatment. The advent of transcatheter aortic valve implantation (TAVI) improved outcomes, with studies suggesting a potential advantage in women compared to men. However, the anatomical differences, such as smaller annuli and more tortuous vascular access, necessitate tailored procedural approaches. Recognizing these sex-specific differences is essential to optimizing AS management, ensuring timely interventions, and improving patient outcomes. Future strategies should incorporate sex-specific thresholds for diagnosis and treatment while leveraging technological advancements, such as artificial intelligence, for personalized therapeutic decisions.

## 1. Epidemiology of Aortic Stenosis in Women

The prevalence of aortic stenosis (AS) increases proportionally with age, rising significantly from 65 years old [1]. Historically, men have been considered at higher risk for severe AS due to their higher prevalence of bicuspid aortic disease. However, recent data show that in a population over 65 years old with valvular disease, women are significantly more prone to AS than men, who are more likely to present aortic regurgitation [2]. Furthermore, although rheumatic diseases significantly decreased nowadays, they can still be a contributing etiology for aortic stenosis.

AS often remains asymptomatic until it reaches a severe stage. Given the substantial morbidity and mortality associated with untreated severe AS, early detection and timely intervention are crucial [3]. At the time of diagnosis, the clinical presentation and baseline characteristics differ between sexes. Women are often diagnosed at a more advanced stage, with more pronounced symptoms despite having fewer comorbidities. The key symptoms reported by women are dyspnea or shortness of breath, along with advanced functional impairment, often categorized as New York Heart Association (NYHA) class III/IV. In contrast, men are more likely to report chest pain [4]. Regarding the comorbidities for patients with severe AS, it has been shown that men have a higher prevalence of coronary artery disease, peripheral vascular disease, and diabetes mellitus, whereas women are more frequently diagnosed with chronic obstructive pulmonary disease (COPD) and hypertension [4,5].

These differences in clinical presentation and comorbidities may be partially attributed to sex-specific anatomical and functional differences and potentially lead to variations in management and outcomes. They may also contribute to a delayed diagnosis in women. Indeed, men, due to a higher prevalence of coronary artery disease, are more frequently referred for cardiac imaging, whereas in women, symptoms may be underestimated or misattributed to non-cardiac causes depending on their overall clinical profile.

## 2. Aortic Stenosis Pathophysiology and Diagnosis

Age-related calcific aortic stenosis is a complex disease driven by mechanical and cellular stress to the aortic valve, leading to sclerosis and subsequent progressive calcification; primary factors initiating damage include mechanical stress and sheer to the endothelial cells on the aortic valve cusps. Such endothelial dysfunction allows for lipid infiltration and oxidation and production of inflammatory mediators which drive cellular signaling within the cusps, resulting in fibrosis and calcification. Significant sex-related differences have been observed in the anatomy, pathophysiology, and progression of AS, influencing both diagnosis and prognosis.

### 2.1. Anatomical Differences

Women generally have a smaller body surface area, which is associated with a smaller aortic annulus, a reduced distance between the annulus and the coronary ostium, a smaller left ventricular outflow tract (LVOT), and a smaller aortic root size [6,7,8]. Regarding the LVOT size in healthy individuals, studies have shown that even after adjusting for body surface area, women still have a smaller LVOT compared to men [6]. These sex-related anatomical differences significantly impact the assessment of AS severity, which relies on echocardiography and assessment of valvular calcification on CT.

### 2.2. Specificities of Echocardiographic Assessment in Women

Paradoxical low-flow, low-gradient AS characterized by an aortic valve area (AVA) ≤ 1.0 cm^2^, a mean gradient < 40 mmHg, a stroke volume index < 35 mL/m^2^, and an LVEF > 50%, is more frequent in women [9,10]. This higher prevalence can be explained by the sex-related anatomical differences.

Indeed, even in healthy individuals, and after adjusting for body size, women tend to have lower stroke volume as well as smaller LVOT area [6,11]. These differences are further exacerbated in the context of the concentric hypertrophy seen in AS. Consequently, women will generally have a lower stroke volume than men at a defined peak velocity across the aortic valve. However, current guidelines recommend using the same threshold to define low SV for both sexes (>35 mL/m^2^), which could contribute to the higher prevalence of paradoxical low-flow low-gradient AS in women. Implementing sex-specific cut-off values (<32 mL/m^2^ for women and <40 mL/m^2^ for men) for defining low-flow, low-gradient aortic stenosis has been shown to improve the ability to assess the prognostic impact of AS [12] and its response to aortic valve replacement [1].

Another important aspect predominantly affecting women is the concept of pressure-recovery: indeed, following rapid flow acceleration across the valve, some of the kinetic energy from the blood flow is converted back to static energy (pressure) in the aortic root. In large aortic roots, the phenomenon of recovery is limited as most of the kinetic energy is dissipated, but in smaller anatomies, this pressure recovery phenomenon might be quite significant, thus reducing the net afterload on the left ventricle. This explains why Doppler echocardiographic measurements of the gradient and aortic valve area, which measures the maximum pressure drop at the level of the valve, tend to be higher than catheterization-derived values that measure the net gradient between the left ventricle and ascending aorta. Doppler thus tends to overestimate the gradient due to pressure recovery [13]. More than two decades ago, Pibarot and colleagues already developed an echocardiographic method taking into account this pressure recovery phenomenon: the energy loss index (ELI) [14]. This concept has been further validated in subsequent studies, showing that adjustment of aortic valve area using the ELI allows for reclassifying the severity of AS in a significant number of patients and that this index provides additional prognostic information compared to conventional measures of AS severity [15,16]. A notable sub-analysis of the SEAS trial confirmed that ELI outperformed conventional indices such as AVAi in predicting adverse valve-related events and all-cause mortality, especially in asymptomatic patients with AS and no known coronary artery disease. Importantly, ELI was shown to be an independent predictor of aortic valve replacement and cardiovascular outcomes. Additionally, the ELI has been associated with higher rates of heart failure-related hospitalization and mortality, even in cases of low-gradient, low-flow AS. While ELI is not incorporated in the guidelines for the evaluation of AS, probably in part due to the relatively limited body of evidence, this remains an additional tool that can be used in borderline cases, especially in the context of small aortic anatomy where the pressure recovery phenomenon is the most important.

When considering prognostic impact and response to treatment of paradoxical low-flow low-gradient AS, the overall body of evidence suggests that paradoxical low-flow low-gradient AS derives a similar benefit from valvular intervention than classical AS [17]. This remains, however, an area of debate and represents a diagnostic challenge that is likely to result in treatment delay that is most likely to affect women for epidemiological reasons [18]. In any case, the condition should not be considered as benign since the mortality rate of paradoxical low-flow low-gradient AS appears at least as high as for classical AS following treatment with TAVI [9].

### 2.3. Left Ventricular Response and Myocardial Fibrosis

The left ventricular (LV) response to chronic pressure overload differs by sex. Men develop larger LV cavities with either concentric or eccentric hypertrophy, whereas women typically exhibit smaller LV cavities with a concentric remodeling pattern [16]. Consequently, the smaller LV cavity and concentric geometry in women result in a reduced SV, with elevated LV filling pressure, more diastolic dysfunction, and a preserved or enhanced LVEF. These ventricular adaptations appear to persist even after the AS has been treated. While some data suggest that women with an adaptative hypertrophic remodeling pattern may experience better survival after AVR compared to men, findings across studies remain inconsistent [19,20]. Furthermore, it has been proposed that this pattern of ventricular response was associated with more fibrosis seen on cardiac magnetic resonance in women than in men, although this has been challenged by more recent data showing a similar amount of diffuse but less focal fibrosis in women than men [21,22].

### 2.4. Valve Calcification and Fibrosis

Valve calcification is a flow-independent measure of AS severity. Calcium load is a key predictor of AS progression and mortality and can be quantified by the Agatston score using non-contrast CT [23]. However, for the same amount of calcium, women tend to experience more severe AS in terms of hemodynamic function than men. This sex-based difference led to the identification of a distinct cutoff value for defining severe aortic stenosis in men (2000 AU) and women (1200 AU) [24]. However, the current approach does not account for interindividual variability in valve size, which may result in the overestimation or underestimation of true AS severity, particularly in patients with large, eccentric annuli (e.g., those with bicuspid aortic valves) or very small annuli (e.g., smaller individuals). indexing aortic valve calcification to valve surface area, as measured by CT, has been proposed as a means to improve the predictive accuracy of AS severity. Integrating this approach into clinical practice could enhance both diagnostic precision and therapeutic decision-making, with a proposed indexed threshold of 300 AU/cm^2^ for women [25].

Beyond calcification, fibrosis plays a crucial role in AS pathophysiology. Histological analyses of resected valves have shown that women exhibit a higher degree of dense connective tissue and fibrosis compared to men with AS of similar severity. This suggests that AS progression in women is primarily driven by fibrotic remodeling, whereas men tend to develop more extensive calcifications [26]. In line with this, assessment of aortic valve fibrocalcific volume using contrast-enhanced CT has been recently shown as a promising tool to assess aortic valve disease severity as well as to potentially predict disease progression, especially in women [27,28]. Indeed, while the correlation between fibrocalcific volume and mean gradient was similar in men and women, the correlation in men was mainly driven by calcific volume, whereas in women, noncalcic volume had a stronger influence. Additionally, women were found to have a significantly higher fibrocalcific ratio compared to men, consistent with the hypothesis of fibrosis playing a larger role in AS progression in women. Further, in women, fibrocalcific volume correlated better with the 1-year disease progression measured by echocardiography than did the standard aortic valve CT calcium score, highlighting the interest of assessing fibrocalcific volume in women and the limitations of traditional calcium-based assessments in this setting.

### 2.5. Disease Progression

Longitudinal studies provided insights into sex-related differences in AS progression. Although the annual increase in transvalvular gradient was similar in women compared to men in the SEAS study [15], the correlation between valve calcification and mean gradient progression was steeper in women. This suggests that even a modest increase in calcification has a greater hemodynamic impact in women. This was also observed in the COFRASA-GENERAC study, where female sex was associated with disease progression [29]. Despite these differences, current evidence remains insufficient to definitively conclude that sex influences the rate of AS progression. When considering evolution of left ventricular function, women with AS tend to maintain better ejection fraction compared to men, which is likely due to their characteristic LV concentric remodeling with an often more diffuse pattern of myocardial fibrosis [30].

### 2.6. Potential Mechanisms Underlying Sex Differences

The mechanisms behind these sex-related differences in AS remain incompletely understood. In addition to anatomical differences, hormonal influences have been associated with increased valvular calcification, with testosterone level positively correlating with vascular calcification and estrogen negatively correlating [31]. While both receptors or such androgen and estrogen are expressed on the aortic valve, further work is needed to understand this direct role on the aortic valve [32]. However, differences in five key pathways are currently proposed to drive sex-specific differences in fibrotic and calcific-mediated stiffening of the aortic valve: the renin–angiotensin–aldosterone system (RAAS), receptor activator of nuclear factor κ B (RANK), Notch, transforming growth factor-β (TGF-β), and Wnt/β-catenin [33]. This could play a key role particularly in postmenopausal women and has been shown to be a factor in AS when studied in those experiencing early menopause and not receiving hormone replacement therapy [34], but further studies are needed to understand the underlying mechanisms [35].

## 3. Treatment

### 3.1. Do Women Have Equal Access to Treatment?

Few studies specifically investigated sex-related differences in AS, as most of the literature focused on outcomes following surgical or transcatheter replacement therapies. Due to the lack of evidence regarding optimal indications and timing for treatment in women, current guidelines recommend the same management approach for both sexes despite differences in anatomy and pathophysiology, but this might change in the next version of the guidelines for the management of valvular heart disease [36]. Adopting a more individualized strategy that incorporates both objective hemodynamic parameters and functional assessments is essential to optimize intervention timing and potentially improve outcomes.

Indeed, recognition of sex-related differences in clinical presentation is important for optimizing AS management since women may be under-diagnosed, as they often present with dyspnea. In contrast, men often present with chest pain and have a higher prevalence of CAD, leading to earlier cardiologic evaluation and earlier AS diagnosis [37].

These diagnostic challenges complicate decision-making regarding the optimal timing for surgical or transcatheter aortic valve replacement (SAVR/TAVI). This seems particularly relevant since there might be a prognostic gain for intervening earlier in women. Indeed, one study looking at the prognostic impact of cardiac damage before TAVI showed that women had a survival advantage over men when they were treated at early stages but that this advantage disappeared at later stages [38].

Several studies have actually shown that women are less frequently referred for aortic valve replacement (AVR) than men, despite experiencing equally severe symptoms [4]. They are also less likely to be evaluated by a cardiac surgeon [39]. A study published in 2010 and conducted at a US university medical center found that three-quarters of patients with severe AS who did not undergo surgery were women [40]. Furthermore, in a cohort of more than 3600 patients with AS, women were 12% less likely than men to receive either surgical or percutaneous AVR [37]. Even after adjusting for age and surgical risk, the AVR rate remained 20% lower in women in another study [39]. A large American registry of over 160,000 patients further corroborated these findings, highlighting greater access to surgery for men compared to women [41]. Within this study, women were also generally found to have worse in-hospital outcomes, including mortality and vascular complications [41]. This is supported by STS database analysis across surgical AVR in the early 2000s in over 100,000 patients that found female sex to have higher operative mortality and stroke [42].

This disparity can be attributed to persistent biases in the epidemiology of AS, as the disease has historically been perceived as predominantly affecting men. Additionally, diagnosing severe AS via echocardiography is often more challenging in women due to more frequent discordant measurements [43] and atypical or underestimated symptoms [40]. Women also tend to be older at the time of diagnosis and present with more frequent multivalvular heart disease [2], leading to greater reluctance toward surgical interventions, especially given that mortality rates are generally less favorable for them [41]. In contrast, men are more likely to have concomitant coronary or aortic disease, which provides an additional rationale for surgery [41].

However, the advent of TAVI over the past two decades reshaped the treatment landscape, offering women a valuable therapeutic alternative [44]. In this context, multidisciplinary heart team discussions are crucial to ensuring optimal individualized management for both women and men.

### 3.2. Management of AS in Women: More Advantages for TAVI?

As mentioned above, surgical outcomes of AVR appear to be less favorable for women than men [41]. Thus, data from two large North American registries, encompassing over 100,000 surgical AVR, indicate that female sex is associated with higher mortality [41] and an increased incidence of stroke [42].

As mentioned above, surgical outcomes AVR appear to be less favorable in women. In this context, TAVI emerged as an appealing alternative, supported by data from the American TVT registry [45] reporting better 1-year survival rates after TAVI in women compared to men, a finding that was further confirmed in a 3-year meta-analysis encompassing >45,000 patients [46]. Moreover, the CoreValve US High Risk Pivotal Trial demonstrated the superiority of TAVI over surgery in women, particularly in terms of mortality and stroke reduction [42].

More recently, the RHEIA trial showed the superiority of TAVI over surgery in women in a randomized study of 443 female patients with severe AS, an average STS score of 2.1%, and a mean age of 73 years, based on a composite primary endpoint of mortality, stroke, and rehospitalizations [47]. In pooled analyses from RHEIA and women within the PARTNER 3 Trial patient population, as presented at Transcatheter Cardiovascular Therapeutics (TCT) 2024, showed that TAVI was also superior to surgical AVR at 1-year follow-up [48]. The difference was mostly driven by a lower rate of rehospitalization in the TAVI group. Additionally, new onset atrial fibrillation and major or life-threatening bleeding were also less frequent in the TAVI group. It must however be noted that longer follow-up will be needed to confirm these early findings. Additionally, TAVI still faces some remaining challenges related to anatomical differences in women, particularly in the management of vascular complications and given the high incidence of small aortic annuli.

### 3.3. TAVI and the Challenge of Female-Specific Anatomical Characteristics

Beyond diagnostic challenges created by the female-specific anatomical characteristics, these later can influence procedural success, complication rates, and long-term outcomes, underscoring the need for a tailored approach to TAVI in women.

Indeed, women tend to have a smaller aortic annulus, with coronary ostia positioned closer to the annulus and narrower aortic sinuses, increasing the risk of annular rupture and coronary occlusion [49,50]. Small aortic annulus, generally defined as an annular area of <400–430 mm^2^, accounts for up to 40% of patients, the vast majority being women [51]. Indeed, a meta-analysis of more than 11,000 patients found that, on average, the aortic annulus diameter is 10% smaller in women [52]. TAVI may allow for a larger effective orifice area and better hemodynamics compared with surgical prostheses given the lack of sewing ring present. The VIVA RCT included 151 patients (93% women) with severe AS and small aortic annuli and noted no differences in MACE at 2 years for TAVI versus SAVR. However, the incidence of severe PPM was almost double in the SAVR group (10.3% vs. 5.6%) despite the use of aortic root enlargements in 7% of patients and newer surgical sutureless valves in 21% of patients in the SAVR group [53].

In line with this, the surgical literature has largely shown that the likelihood of implanting a small bioprosthesis is much higher in women. As a consequence, female sex has been identified as a risk factor for severe patient–prosthesis mismatch after SAVR [54,55], a condition associated with a worse long-term prognosis. In contrast, it appears that this risk can be mitigated in the context of TAVI, thus offering another potential advantage over surgery in women.

To further clarify the impact of transcatheter heart valve design on outcomes in the context of small annulus, the multicenter SMART trial included 716 severe AS patients with small aortic annuli, of which 87% were women and were randomized to a self-expanding valve or balloon-expandable valve [56]. A recent post hoc analysis of the trial reported better hemodynamic outcomes and a lower mismatch rate with a self-expandable valve in the women subgroup [57]. Additionally, the combination of smaller annuli, combined with less calcified valves, offers an advantage in terms of reducing the incidence of paravalvular leak, a factor clearly associated with a negative prognostic impact [58].

Vascular access also differs in women, as they tend to have smaller, more calcified, and more tortuous iliofemoral arteries [52]. This anatomical difference results in a smaller femoral artery-to-delivery system diameter ratio, which might explain the higher incidence of vascular complications in women that has been observed in several studies, particularly when BMI is low [59]. Since the available evidence on the topic comes from studies using data collected several years ago, one could wonder whether the advent of the latest generation of low-profile delivery systems as well as improved use of multimodality imaging for TAVI planification and execution narrowed this difference between men and women [60]. However, recent data from VIVA and RHEIA looking at TAVI in women suggest that event rate has not dramatically decreased and that women might still be at a higher risk of vascular complication. These findings underscore the importance of a thorough pre-TAVI CT scan analysis to identify high-risk anatomical features and minimize complications [61].

In terms of conduction disturbances, women have a 10% lower risk of requiring permanent pacemaker implantation compared to men [62]. This may be attributed to the distribution of valve calcifications in women, which tend to spare the non-coronary cusp, the cusp most closely associated with conduction pathways. Interestingly, the benefit appears most prominent when self-expanding valves are used, while the difference seems non-significant for balloon-expandable valves, thus offering another element in favor of the use of self-expanding platforms in women [62].

Finally, in case of implantation of a bioprosthetic valve, whether surgical or transcatheter, long-term valve function and degeneration needs to be considered in the overall lifetime management of women with aortic stenosis. While few studies considered this, since women tend to receive smaller bioprosthetic valves, this may impact reintervention in terms of subsequent patient–prosthesis mismatch and risk of coronary occlusion. Some data from the PARTNER-2 trial suggest that female sex was associated with an increased incidence in structural valve deterioration over time both in the surgical and transcatheter groups [63]. However, since the implantation of a smaller bioprosthesis was more frequent in women and was also associated with structural valve deterioration, it remains unclear whether there was an interaction between sex and valve size or whether sex is truly an independent factor due to some underlying hormonal pathophysiological effect. Thus, long-term follow-up from low-risk trials as well as the RHEIA and SMART trials will be crucial to refine our understanding of the impact of sex, valve type (balloon-expandable versus self-expanding), and mode of implant (TAVI versus surgery) on long-term valve function.

## 4. Conclusions

**Figure 1:**
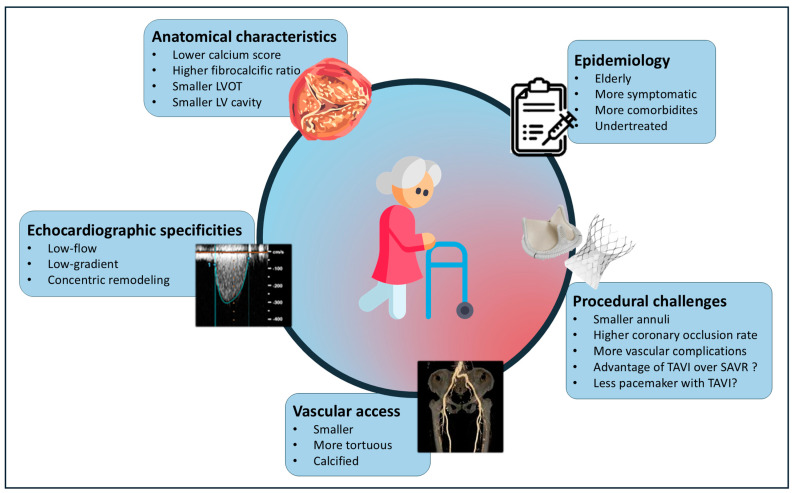
Specificities and challenges of aortic stenosis in women. LVOT: left ventricular outflow tract. LV: left ventricle. TAVI: transcatheter aortic valve implantation. SAVR: surgical aortic valve replacement.

AS in women presents distinct diagnostic and therapeutic considerations that warrant a re-evaluation of current management strategies. A personalized approach, incorporating dedicated standards for women, should be implemented, from echocardiographic diagnosis to the selection of the treatment modality (SAVR versus TAVI), as well as the choice of the TAVI prosthesis and pre-procedural CT scan analysis (Figure 1). More broadly, these sex-specific differences should prompt a reconsideration of our approach to all valvular heart diseases, not just AS.

## Data Availability

Not applicable.
